# Association of the Preoperative CALLY Index with Acute Kidney Injury After Cytoreductive Surgery and Hyperthermic Intraperitoneal Chemotherapy: A Comparison with Other Inflammatory and Immune-Nutritional Indices

**DOI:** 10.3390/jcm15187308

**Published:** 2026-09-20

**Authors:** Hatice Şahin, Gülay Ulusal Okyay, Fatma Ayerden Ebinç, Berrak Itır Aylı, Fevzi Coşkun Sökmen, Ferit Aydın, Bülent Aksel, Mehmet Deniz Aylı

**Affiliations:** 1 Division of Nephrology, Department of Internal Medicine, Ankara Etlik City Hospital, Ankara 06170, Türkiye; mdgulayulusal@hotmail.com (G.U.O.); fayerdenebin@yahoo.com (F.A.E.); d_ayli@hotmail.com (M.D.A.); 2 School of Life Sceinces, University of Westminster, London W1B 2HW, UK; itirayli@gmail.com; 3 Department of Internal Medicine, Gulhane Faculty of Medicine, University of Health Sciences, Ankara 06010, Türkiye; fcoskunsokmen@gmail.com; 4 Department of Surgical Oncology, Ankara Etlik City Hospital, Ankara 06170, Türkiye; drferita@gmail.com (F.A.); akselbulent@gmail.com (B.A.)

**Keywords:** acute kidney injury, hyperthermic intraperitoneal chemotherapy, cytoreductive surgery, CALLY index, immune-nutritional biomarkers, inflammatory indices, cisplatin

## Abstract

**Background/Objectives:** Acute kidney injury (AKI) is a frequent complication following cytoreductive surgery with hyperthermic intraperitoneal chemotherapy (CRS-HIPEC). The association between preoperative inflammatory and immune-nutritional indices and postoperative AKI after CRS-HIPEC remains unclear. We aimed to investigate whether these prespecified candidate preoperative markers are associated with postoperative AKI in patients undergoing CRS-HIPEC. **Methods:** This retrospective single-center study included 87 patients who underwent CRS-HIPEC between October 2022 and April 2025. All received intraoperative cisplatin and mitomycin C. AKI was defined according to Kidney Disease: Improving Global Outcomes serum creatinine criteria. Preoperative neutrophil-to-lymphocyte ratio, platelet-to-lymphocyte ratio, systemic immune-inflammation index, pan-immune-inflammation value, systemic inflammation response index, and C-reactive protein–albumin–lymphocyte (CALLY) index were calculated from routine laboratory parameters. Each candidate marker was evaluated in a separate model adjusted for age and sex, and then in a second model additionally adjusted for baseline estimated glomerular filtration rate. Discriminatory performance was assessed using receiver operating characteristic analysis. The CALLY index was calculated using albumin in g/dL, lymphocyte count in ×10^9^/L, and C-reactive protein in mg/L. **Results:** AKI developed in 24 patients (27.6%). In-hospital mortality was higher in patients with AKI than in those without AKI (25.0% vs. 1.6%; *p* = 0.002). A higher log10-transformed CALLY index remained associated with lower odds of AKI after adjustment for age and sex (adjusted OR, 0.276; 95% CI, 0.113–0.673; *p* = 0.005). The CALLY index had the numerically highest AUC among the evaluated indices, with moderate discriminatory performance (AUC, 0.708; 95% CI, 0.579–0.836). A data-derived exploratory cutoff of ≤1.640 yielded 79.2% sensitivity and 63.5% specificity. **Conclusions:** In this selected retrospective cohort of patients without established chronic kidney disease, a lower preoperative CALLY index was associated with postoperative AKI and showed moderate discrimination as an individual marker. The data-derived threshold should be considered exploratory and requires independent validation before clinical application.

## 1. Introduction

Cytoreductive surgery and hyperthermic intraperitoneal chemotherapy (CRS-HIPEC) have emerged as an important treatment option for selected patients with peritoneal surface malignancies. Despite its oncologic benefits, CRS-HIPEC is associated with substantial perioperative morbidity and mortality [1,2,3]. Renal complications are of special concern, as they may complicate postoperative recovery and negatively influence short- and long-term outcomes [3,4,5,6,7].

Acute kidney injury (AKI) is among the most clinically important complications of CRS-HIPEC, with reported incidence rates ranging from 1% to 48%, reflecting differences in patient selection, chemotherapy regimens, perioperative management, and AKI definitions [3,4,5,6,8,9,10,11,12,13]. Renal injury in this setting is usually multifactorial. Cisplatin can cause direct tubular toxicity through oxidative stress, mitochondrial dysfunction, inflammation, and DNA damage [5,12,14]. In addition, hyperthermia-induced vasodilation, increased intra-abdominal pressure, major fluid shifts, blood loss, prolonged surgery, vasopressor use, and exposure to other nephrotoxic agents may impair renal perfusion and contribute to ischemic tubular injury [6,10,15].

Several clinical and perioperative risk factors for AKI after CRS-HIPEC have been reported, including older age, obesity, lower baseline estimated glomerular filtration rate (eGFR), chronic kidney disease (CKD), cisplatin-based HIPEC, higher peritoneal cancer index (PCI), hypoalbuminemia, blood loss, nephrotoxic antibiotic exposure, longer intensive care unit (ICU) stay, and use of renin–angiotensin system (RAS) blockers [4,5,6,8,9,10,11,12,13]. However, these associations have been inconsistent across studies, and AKI may occur despite apparently preserved preoperative kidney function. This suggests that conventional clinical and renal parameters may not fully capture perioperative renal vulnerability in this setting. Therefore, simple and accessible preoperative biomarkers may help improve AKI risk stratification in this high-risk surgical population.

Systemic inflammatory and immune-nutritional indices such as the neutrophil-to-lymphocyte ratio (NLR), platelet-to-lymphocyte ratio (PLR), systemic immune-inflammation index (SII), pan-immune-inflammation value (PIV), systemic inflammation response index (SIRI), and C-reactive protein–albumin–lymphocyte (CALLY) index are derived from routine blood tests and reflect systemic inflammation, immune status, and nutritional reserve. Because these biological domains may contribute to perioperative organ vulnerability, such indices may represent clinically accessible candidate preoperative markers. Although these indices have shown clinical relevance in surgical and oncologic populations [16,17,18,19,20], their association with postoperative AKI after CRS-HIPEC has not yet been investigated.

This study aimed to evaluate the association between preoperative inflammatory and immune-nutritional indices and postoperative AKI in patients undergoing CRS-HIPEC with cisplatin and mitomycin C. Secondary objectives were to examine the incidence, severity, clinical consequences and perioperative factors associated with AKI in this cohort.

## 2. Materials and Methods

### 2.1. Study Design and Population

This retrospective observational study was approved by the Ethics Committee of Etlik City Hospital, Ankara, Türkiye (24 June 2025; approval no. AEŞH-BADEK2-2025-138). The requirement for informed consent was waived due to the retrospective design. The study was conducted in accordance with the Declaration of Helsinki.

Patients who underwent CRS-HIPEC at our institution between 1 October 2022, and 1 April 2025, were retrospectively screened. All procedures were performed by the same surgical team. Eligible patients were 18–80 years of age, had an American Society of Anesthesiologists (ASA) physical status of I–III, an Eastern Cooperative Oncology Group (ECOG) performance status of 0–2, had histologically confirmed peritoneal carcinomatosis, and achieved a completeness of cytoreduction (CC) score of CC-0 or CC-1. For patients who had previously received systemic chemotherapy or chemoradiotherapy, an interval of at least three weeks between the last treatment session and CRS-HIPEC was required. Patients were excluded if clinical or laboratory data were incomplete, including missing serum creatinine measurements at any of the predefined perioperative time points (preoperative baseline and postoperative days 1, 2, 3, 5, and 7), or if they had pre-existing CKD, defined as an eGFR < 60 mL/min/1.73 m^2^ for at least three months. To avoid double counting and ensure patient-level independence, patients who underwent more than one CRS-HIPEC procedure during the study period were included only once in the analysis, with data from their first procedure used for analysis.

### 2.2. Data Collection

Demographic and baseline clinical variables included age at surgery, sex, body mass index (BMI), body surface area, comorbidities, primary tumor type, ASA physical status, ECOG performance status, and PCI. Recorded comorbidities included hypertension, diabetes mellitus, and coronary artery disease. Preoperative use of RAS blockers was documented. Data on previous systemic chemotherapy and prior exposure to platinum-based regimens were also collected.

Operative and HIPEC-related variables included the date of surgery, cisplatin and mitomycin C doses, perfusate volume, HIPEC duration and temperature, and intraoperative vasopressor use. Postoperative data included invasive mechanical ventilation, aminoglycoside exposure, intravenous contrast administration, renal replacement therapy (RRT), postoperative complications, ICU and hospital length of stay, and in-hospital mortality. The timing of postoperative variables was also recorded. Postoperative complications were defined as the occurrence of at least one of the following: bleeding, wound infection, anastomotic leakage, intra-abdominal abscess, evisceration, or bladder fistula.

### 2.3. Laboratory Measurements and Inflammatory Indices

Preoperative laboratory measurements were obtained from blood samples collected within seven days before surgery. Complete blood count parameters, including white blood cell, neutrophil, lymphocyte, monocyte, and platelet counts and hemoglobin concentration, were measured using a Sysmex XN-1000 hematology analyzer (Sysmex Corporation, Kobe, Japan). Serum creatinine was measured by the Jaffé method using a Roche Cobas c702 analyzer (Roche Diagnostics, Mannheim, Germany), and eGFR was calculated using the 2021 CKD Epidemiology Collaboration creatinine equation [21]. Serum albumin, C-reactive protein (CRP), glucose, urea, sodium, potassium, and calcium levels were measured using routine automated laboratory methods.

Six preoperative systemic inflammatory and immune-nutritional indices were calculated from baseline laboratory values: NLR, PLR, SII, PIV, SIRI, and the CALLY index. Neutrophil, lymphocyte, monocyte, and platelet counts were expressed as ×10^9^/L. CRP was expressed as mg/L and albumin as g/dL. The indices were calculated as follows: NLR = neutrophil count/lymphocyte count; PLR = platelet count/lymphocyte count; SII = platelet count × neutrophil count/lymphocyte count; PIV = neutrophil count × platelet count × monocyte count/lymphocyte count; SIRI = neutrophil count × monocyte count/lymphocyte count; and CALLY index = albumin (g/dL) × lymphocyte count (×10^9^/L, numerically equivalent to ×10^3^/µL)/CRP (mg/L). Because CALLY values depend on the measurement units used for its components, the reported cutoff applies to calculations based on albumin in g/dL, lymphocyte count in ×10^3^/µL (equivalent to ×10^9^/L), and CRP in mg/L.

### 2.4. Study Outcomes, Definition and Staging of AKI

The primary outcome was the development of AKI within the first seven days after CRS-HIPEC. Secondary outcomes included AKI stage, need for RRT during hospitalization, postoperative complications, ICU and hospital length of stay, and in-hospital mortality. Serum creatinine was recorded preoperatively and on postoperative days 1, 2, 3, 5, and 7. Baseline serum creatinine was defined as the most recent value obtained within seven days before surgery. Baseline eGFR was calculated from the preoperative serum creatinine value.

AKI was defined and staged according to the serum creatinine criteria of the Kidney Disease: Improving Global Outcomes (KDIGO) guideline. AKI was diagnosed as an increase in serum creatinine of ≥0.3 mg/dL within 48 h or to ≥1.5 times the baseline value within seven days after surgery. Stage 1 AKI was defined as an increase of ≥0.3 mg/dL within 48 h or to 1.5–1.9 times baseline. Stage 2 AKI was defined as an increase to 2.0–2.9 times baseline, whereas stage 3 AKI was defined as a rise in serum creatinine to ≥3.0 times baseline, a serum creatinine level of ≥4.0 mg/dL, or initiation of RRT [22]. Urine output criteria were not applied because complete and reliable urine output data were not available for all patients. For comparative analyses, patients were classified according to the development of AKI within the first seven postoperative days.

### 2.5. Institutional CRS-HIPEC Protocol and Perioperative Management

All patients underwent preoperative anesthetic evaluation. RAS blockers were discontinued 48 h before surgery. Following the necessary cytoreductive surgery, four drains were inserted for the HIPEC procedure. Before HIPEC, patients received standard intraoperative hemodynamic and fluid management, including a 1 L bolus of 0.9% saline unless contraindicated. HIPEC was administered at 42 °C for 60 min through the closed-abdomen technique using the ThermoChem™ HT-2500 device (ThermaSolutions, Breda, The Netherlands). Normal saline was used as the carrier solution. The cisplatin dose was calculated according to the following formula: body surface area (m^2^, calculated using the Mosteller formula) × 25 mg/m^2^ × perfusate volume (L). No dose fractionation or predefined maximum dose of cisplatin was used. Mitomycin C was administered intraperitoneally at a standard dose of 20 mg without dose fractionation. After surgery, patients were monitored in the ICU for 24–48 h. Intravenous hydration was maintained at approximately 3–4 L/day, adjusted according to clinical status and contraindications, with a target urine output of at least 100 mL/hour. The hydration and urine-output protocol remained unchanged throughout the study period. Sodium thiosulfate, magnesium, mannitol, intravenous furosemide, and other specific nephroprotective strategies were not routinely administered.

### 2.6. Statistical Analysis

The distribution of continuous variables was assessed using the Shapiro–Wilk test and visual inspection. Normally distributed variables were presented as mean ± standard deviation (SD) and compared using the independent-samples Student’s *t*-test. Non-normally distributed variables were presented as median with interquartile range (IQR) and were compared using the Mann–Whitney U test. Categorical variables were presented as counts and percentages and compared using the chi-square or Fisher’s exact test, as appropriate. Univariable logistic regression was used to evaluate associations with postoperative AKI. For additional clinical and laboratory covariates, variables with a *p*-value < 0.10 in either the between-group comparisons or univariable analyses were considered for exploratory adjusted analyses. In contrast, NLR, PLR, SII, PIV, SIRI, and the CALLY index were evaluated as a predefined group of study-specific candidate markers irrespective of univariable statistical significance. Age and sex were selected on clinical grounds as relevant demographic covariates, and baseline eGFR was additionally evaluated in a separate covariate-adjusted model. Model 1 included the variable of interest, age, and sex, whereas Model 2 additionally included baseline eGFR. Given the limited number of AKI events (n = 24), all adjusted analyses were considered exploratory. Variables producing complete separation were not entered into standard logistic regression models and were reported descriptively. Adjusted odds ratios (ORs) for the variable of interest were reported with 95% confidence intervals (CIs). For continuous variables, ORs were reported according to specified increments: lymphocyte count per 0.1 ×10^9^/L, PLR per 10 units, NLR per 1 unit, CRP per 1 mg/L, SII and PIV per 100 units, and SIRI per 1 unit. Perfusate volume was analyzed per 1 mL increase.

Because of the right-skewed distribution of the CALLY index, CALLY was log10-transformed for logistic regression analyses. ORs therefore represent the change in the odds of AKI per 1-unit increase in log10 (CALLY), corresponding to a 10-fold increase in CALLY on its original scale. The same transformation was used consistently in univariable and adjusted regression models.

Model fit was assessed using the Akaike Information Criterion (AIC) and McFadden’s pseudo-R^2^. Discriminatory performance was evaluated using receiver operating characteristic (ROC) curve analysis. Areas under the curve (AUCs) and 95% CIs were estimated using the DeLong method, and exploratory threshold values were determined by maximizing the Youden index. ROC analyses and threshold determination for the CALLY index were performed using the original, untransformed CALLY values. For variables in which lower values indicated greater AKI risk, including CALLY, lymphocyte count, and eGFR, the direction of classification was reversed accordingly. AUCs were compared pairwise using the DeLong test, with *p*-values adjusted for multiple comparisons using the Holm procedure.

All statistical tests were two-sided, and a *p*-value < 0.05 was considered statistically significant. Statistical analyses were performed using Python version 3.12 with the following packages: pandas (v2.2) for data management, scipy (v1.14) for statistical tests, statsmodels (v0.14) for logistic regression analysis, and scikit-learn (v1.5) for ROC curve analysis.

## 3. Results

**Figure 1:**
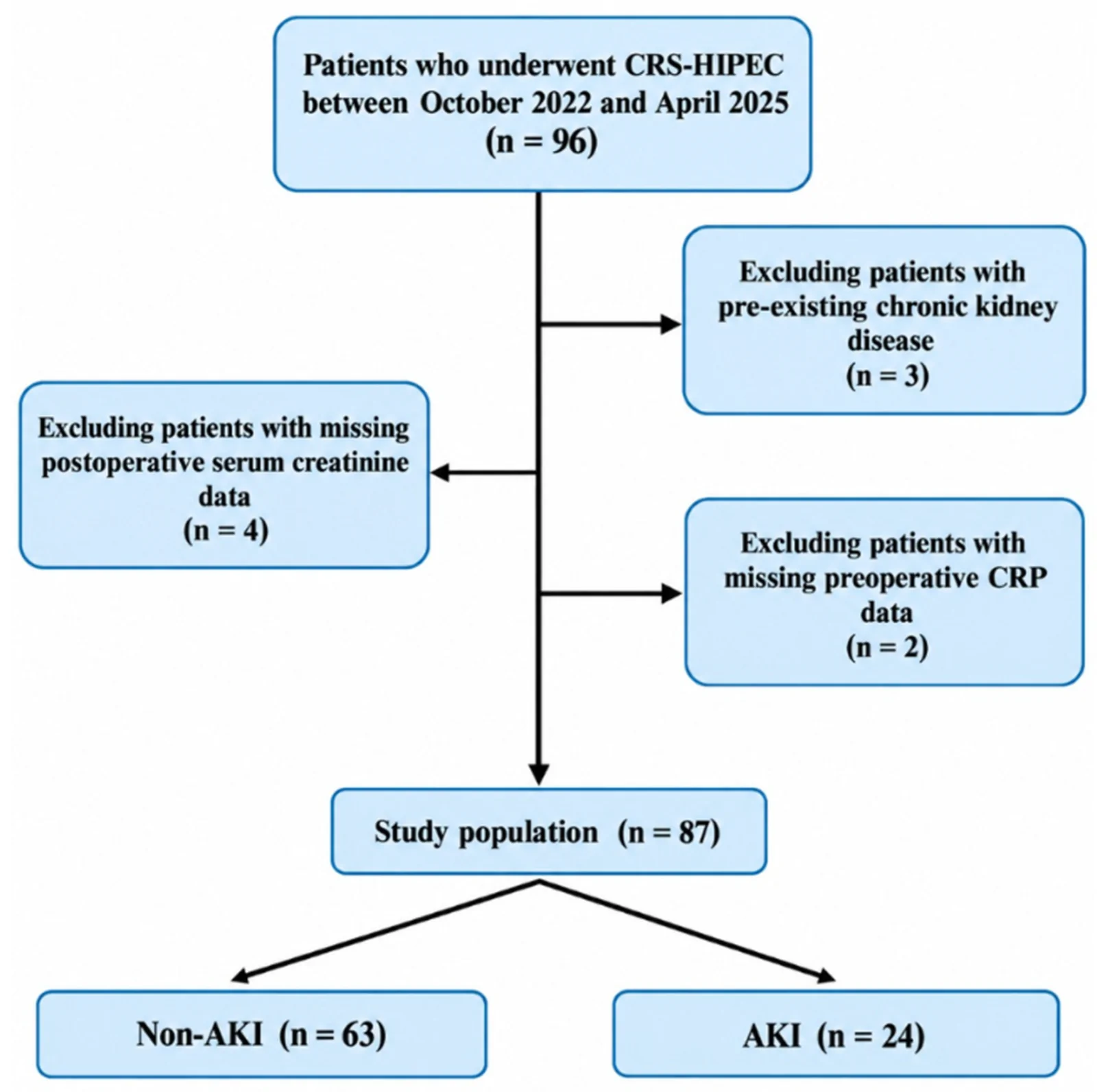
Study flowchart. Of 96 screened patients, 87 were retained for the final analysis after excluding 4 patients with missing postoperative serum creatinine data, 3 patients with pre-existing chronic kidney disease, and 2 patients with missing preoperative C-reactive protein (CRP) data. Among the 4 patients who underwent repeat CRS-HIPEC procedures, only data from the first procedure were included in the analysis.

During the study period, 96 patients were screened. All patients received cisplatin plus mitomycin C. After excluding six patients with incomplete data and three with pre-existing CKD, 87 patients were included in the final analysis. Among the four patients who underwent repeated CRS-HIPEC, only data from the first CRS-HIPEC procedure were analyzed. The study flowchart is shown in Figure 1.

**Figure 2:**
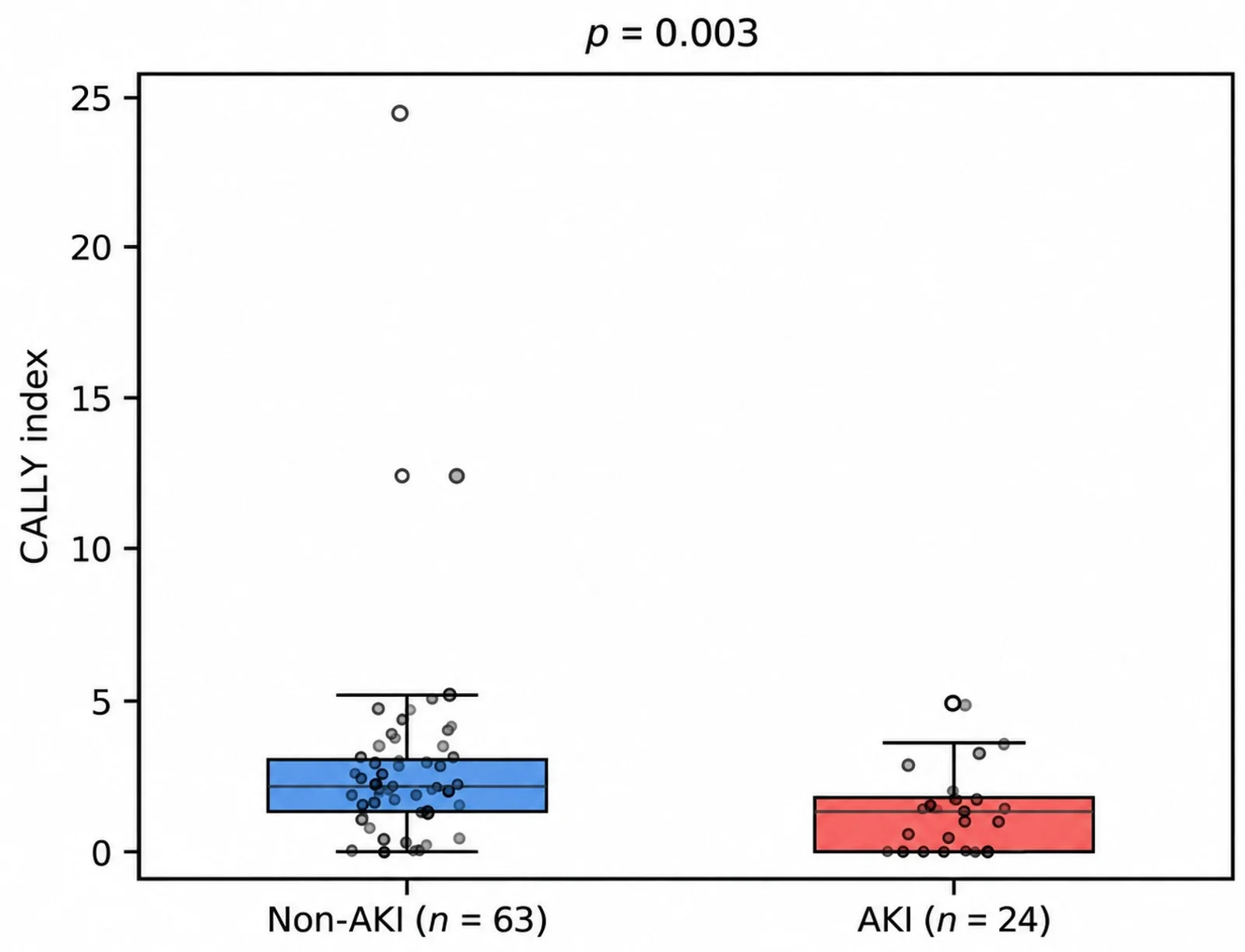
Distribution of the preoperative CALLY index according to postoperative acute kidney injury (AKI) status. The CALLY index differed between the non-AKI (n = 63) and AKI (n = 24) groups (*p* = 0.003, Mann–Whitney U test). The circles indicate individual data points. CALLY, C-reactive protein–albumin–lymphocyte index.

Baseline demographic, clinical, oncologic, and laboratory characteristics of the study cohort and subgroups are presented in Table 1 and Table 2. The median age was 60 years (IQR, 48–69), 65 patients (74.7%) were female, and the most common primary malignancies were colorectal cancer (31.0%) and ovarian cancer (28.7%). Postoperative AKI developed in 24 patients (27.6%). No significant between-group differences were observed in age, sex, BMI, body surface area, primary malignancy, ASA physical status, ECOG performance status, or PCI. Hypertension was more common in the AKI group than in the non-AKI group (66.7% vs. 38.1%; *p* = 0.032). Patients who developed AKI had lower preoperative hemoglobin levels (10.46 ± 2.13 vs. 11.61 ± 1.78 g/dL; *p* = 0.025) and lymphocyte counts (1.16 [IQR, 1.00–1.63] vs. 1.66 [IQR, 1.15–2.05] × 10^9^/L; *p* = 0.022). Among the composite indices, CALLY was lower in the AKI group (1.33 [IQR, 0.06–1.61] vs. 2.09 [IQR, 1.28–2.94]; *p* = 0.003), whereas NLR was higher (4.88 [IQR, 1.92–7.15] vs. 2.62 [IQR, 1.88–3.73]; *p* = 0.042). CRP also differed between groups (*p* = 0.049). Postoperative characteristics and outcomes according to AKI status are shown in Table 3. In two patients, the need for postoperative mechanical ventilation developed before the onset of AKI. AKI was observed in 3 of the 4 patients who received aminoglycosides, and in all four patients, aminoglycoside therapy was administered more than one week after AKI onset. Among the 14 patients who underwent contrast-enhanced computed tomography, AKI occurred in 5 patients. None of these five patients underwent contrast-enhanced computed tomography within the first 7 days after surgery. Of the 24 patients with AKI, 12 (50.0%) had stage 1, four (16.7%) had stage 2, and eight (33.3%) had stage 3 AKI. RRT was required in four patients, all of whom had stage 3 AKI. Hospital length of stay differed across AKI stages, with median durations of 12, 15.5, and 33 days for stages 1, 2, and 3, respectively (*p* = 0.017). All patients with stage 3 AKI experienced at least one postoperative complication. In-hospital mortality was 75.0% in the stage 3 group, compared with 1.6% in the non-AKI group; no deaths occurred in patients with stage 1 or stage 2 AKI. The causes of death were postoperative complications and sepsis-related multiorgan failure. No patients died within the first 7 postoperative days. In the AKI group, the median time to peak serum creatinine was postoperative day 3 (Table 4). The distribution of the CALLY index according to AKI status is shown as a box plot in Figure 2.

### 3.1. Univariable Logistic Regression Analysis

In univariable logistic regression analyses (Table 5), a higher CALLY index was associated with lower odds of postoperative AKI (OR, 0.591; 95% CI, 0.409–0.854; *p* = 0.005). Conversely, higher PLR (OR, 1.029; 95% CI, 1.002–1.058; *p* = 0.037) and SII (OR per 100-unit increase, 1.041; 95% CI, 1.006–1.076; *p* = 0.020) were associated with higher odds of AKI. Among the individual clinical and laboratory variables, higher hemoglobin (OR per 1 g/dL increase, 0.734; 95% CI, 0.569–0.946; *p* = 0.017), lymphocyte count (OR, 0.901; 95% CI, 0.814–0.997; *p* = 0.043), and albumin (OR per 1 g/dL increase, 0.450; 95% CI, 0.218–0.929; *p* = 0.031) were associated with lower odds of AKI. Higher CRP (OR per 1 mg/L increase, 1.022; 95% CI, 1.000–1.044; *p* = 0.047) and hypertension (OR, 3.250; 95% CI, 1.208–8.741; *p* = 0.020) were associated with higher odds of AKI. Age, BMI, diabetes mellitus, baseline serum creatinine, eGFR, PCI, cisplatin dose, and previous platinum exposure were not significantly associated with postoperative AKI. An OR could not be estimated for intraoperative vasopressor use because all four patients who required vasopressor support developed AKI, resulting in complete separation.

### 3.2. Age- and Sex-Adjusted, and Age-, Sex-, and Baseline eGFR-Adjusted Logistic Regression Analyses

In the age- and sex-adjusted analyses, each candidate variable was examined in a separate logistic regression model that included age and sex as covariates (Table 5). A higher CALLY index remained associated with lower odds of postoperative AKI (adjusted OR, 0.276; 95% CI, 0.113–0.673; *p* = 0.005). Among the evaluated inflammatory and immune-nutritional indices, the CALLY model showed the lowest AIC and the highest McFadden pseudo-R^2^ (AIC, 100.012; pseudo-R^2^, 0.1022; Supplementary Table S1). PLR, SII, NLR, and PIV also remained associated with AKI after adjustment for age and sex. Higher hemoglobin was associated with lower odds of AKI, whereas higher CRP and hypertension were associated with higher odds. The association between lymphocyte count and AKI was of borderline statistical significance (adjusted OR, 0.903; 95% CI, 0.815–1.001; *p* = 0.053).

**Figure 3:**
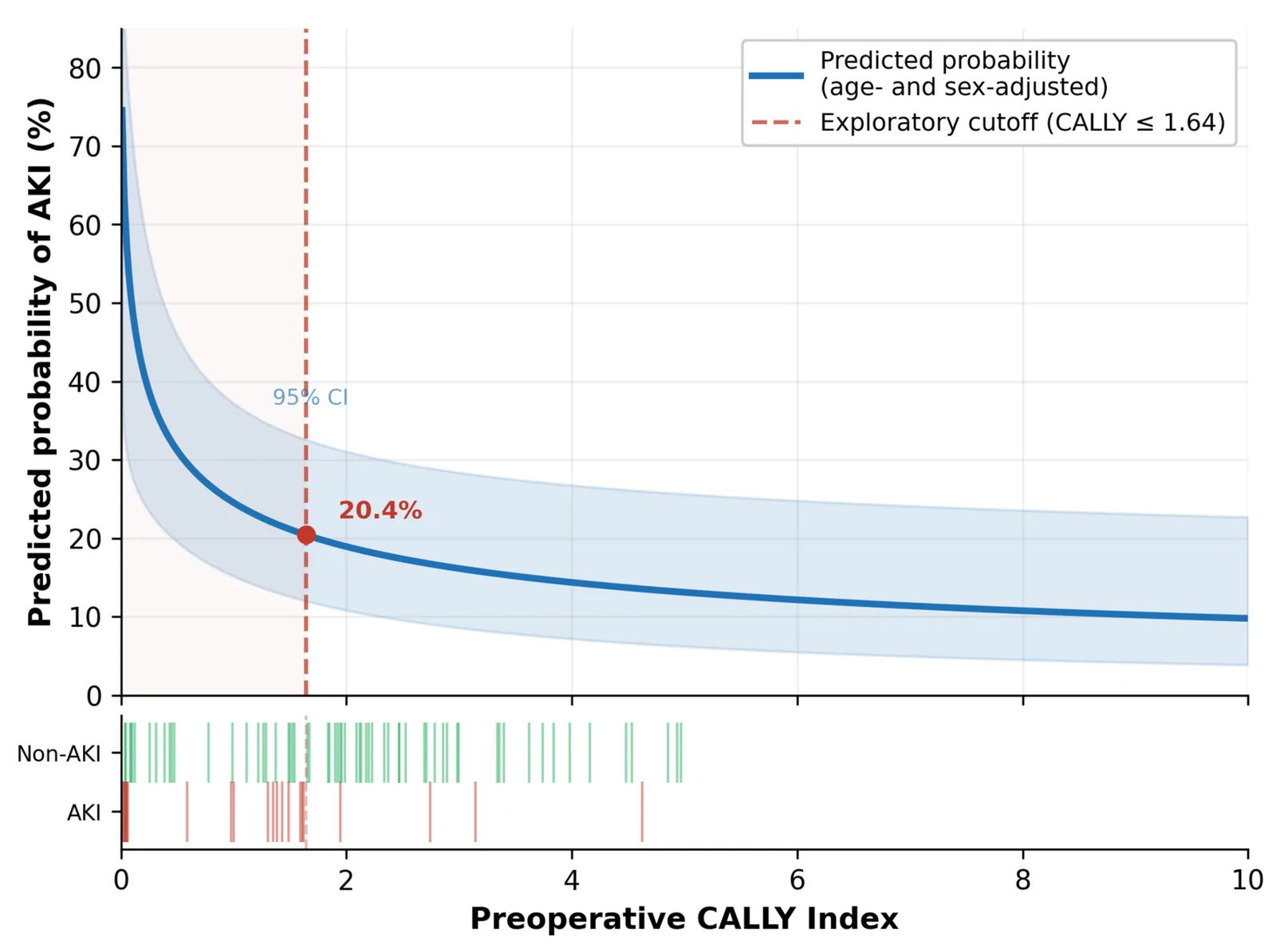
Predicted probability of postoperative AKI (acute kidney injury) as a function of the preoperative CALLY (C-reactive protein–albumin–lymphocyte) index, derived from the age- and sex-adjusted logistic regression model (covariates held at mean age and female sex). The blue shaded area represents the 95% confidence interval. The vertical dashed line indicates the exploratory cutoff of ≤1.640. Individual patient CALLY values are shown as a rug plot at the bottom, stratified by AKI status (red = AKI; green = non-AKI).

The findings were broadly similar after additional adjustment for baseline eGFR (Table 5). CALLY was evaluated as a continuous log10-transformed variable in all regression analyses. The predicted probability of postoperative AKI derived from the age- and sex-adjusted model decreased gradually with increasing CALLY values, without evidence of a discrete threshold effect (Figure 3). The CALLY index remained associated with lower odds of AKI (adjusted OR, 0.283; 95% CI, 0.116–0.691; *p* = 0.006). PLR, SII, PIV, hemoglobin, and hypertension also remained significantly associated with AKI. In contrast, the associations of NLR and CRP were attenuated and no longer reached statistical significance. Lymphocyte count remained at the conventional threshold of significance (adjusted OR, 0.901; 95% CI, 0.811–1.000; *p* = 0.050) (Table 5).

Because of the limited number of AKI events (n = 24), each candidate variable was evaluated in a separate logistic regression model adjusted for age and sex rather than in a single comprehensive multivariable model. These exploratory adjusted analyses therefore provide covariate-adjusted association estimates for individual variables and should not be interpreted as a comprehensive multivariable prediction model.

### 3.3. Receiver Operating Characteristic Analysis

**Figure 4:**
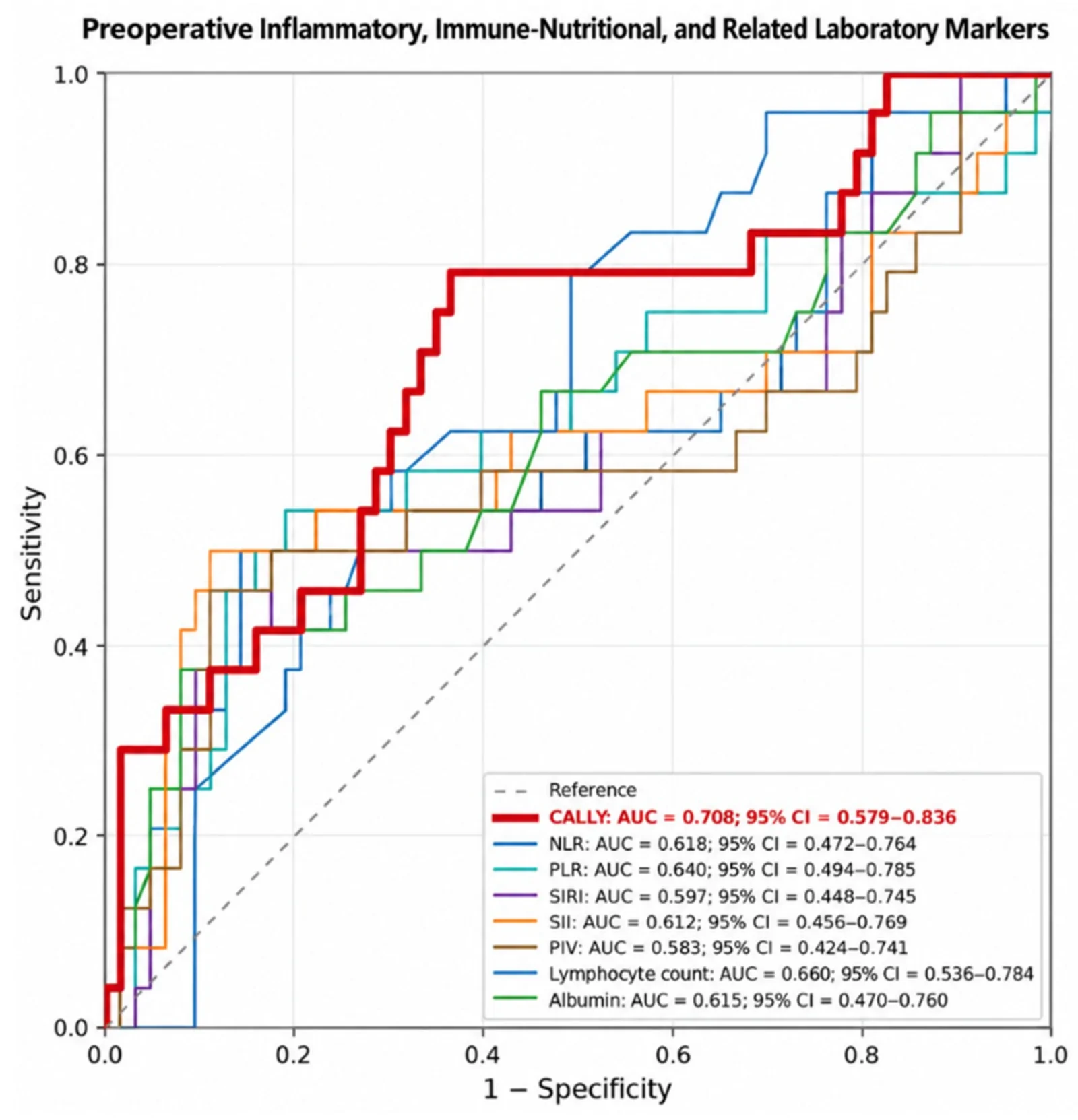
Receiver operating characteristic curves of preoperative inflammatory and immune-nutritional indices, albumin, and lymphocyte count for discriminating postoperative AKI. The CALLY index was oriented so that lower values indicated a higher risk of postoperative AKI. Data-derived exploratory cutoffs were determined using the maximum Youden index and are reported in Table 6. The dashed diagonal line represents an AUC of 0.50. Abbreviations: AKI, acute kidney injury; AUC, area under the curve; CALLY, C-reactive protein–albumin–lymphocyte index; CI, confidence interval; NLR, neutrophil-to-lymphocyte ratio; PIV, pan-immune-inflammation value; PLR, platelet-to-lymphocyte ratio; SII, systemic immune-inflammation index; SIRI, systemic inflammation response index.

ROC analyses of the inflammatory and immune-nutritional indices are presented in Figure 4 and Table 6. Among the evaluated variables, the CALLY index showed the numerically highest AUC for postoperative AKI, with an AUC of 0.708 (95% CI, 0.579–0.836). A cutoff of ≤1.640 yielded 79.2% sensitivity and 63.5% specificity, corresponding to a Youden index of 0.427. Lymphocyte count showed modest discrimination, with an AUC of 0.660 (95% CI, 0.536–0.784). A cutoff of ≤1.630 yielded 79.2% sensitivity and 50.8% specificity. Among the remaining composite indices, PLR had the next highest AUC (0.640; 95% CI, 0.494–0.785), followed by NLR, albumin, SII, SIRI, and PIV. In contrast, baseline eGFR (AUC, 0.541; 95% CI, 0.393–0.689) and serum creatinine (AUC, 0.494; 95% CI, 0.348–0.640) showed limited discriminatory performance. Pairwise comparisons of the AUCs were performed using the DeLong test, with Holm adjustment for multiple comparisons. The results of these comparisons are presented in Supplementary Table S2.

## 4. Discussion

This study evaluated the association between preoperative inflammatory and immune-nutritional indices and postoperative AKI in patients undergoing CRS-HIPEC with cisplatin and mitomycin C. AKI developed in more than one-quarter of the cohort and was associated with more postoperative complications, longer ICU and hospital stays, and higher in-hospital mortality. Baseline serum creatinine and eGFR did not differ between patients with and without AKI. In contrast, a lower preoperative CALLY index remained associated with AKI after adjustment for age, sex, and baseline kidney function and showed the numerically highest, although moderate, discriminatory performance among the evaluated indices. To our knowledge, this is the first study to compare a broad panel of preoperative inflammatory and immune-nutritional indices with respect to their association with AKI after CRS-HIPEC.

AKI remains one of the most frequent and clinically relevant complications of CRS-HIPEC, with substantial variation in reported incidence across studies [3,4,5,6,8,9,10,11,12]. A 2024 systematic review of 1622 patients reported a pooled incidence of 23.4%, increasing to 34.7% in cisplatin-based protocols [2]. Among individual cohorts, Arjona-Sánchez et al. reported an incidence of 30.5% [9], compared with 27.6% in our cohort and 21.5% in the study by Lu et al. [6]. Lower rates were reported by Carias et al. [4], Chen et al. [10], and Beltrán et al. [12], whose cohorts included patients treated with mitomycin C alone or other non-platinum regimens. This variability likely reflects differences in patient selection, chemotherapy regimens, perioperative fluid and hemodynamic management, nephroprotective strategies, and AKI definitions. Nevertheless, the consistently substantial incidence across contemporary studies confirms that AKI is a major complication of CRS-HIPEC. Cisplatin may have further increased this risk, as all patients in our cohort received cisplatin. Similarly, Tan et al. reported a higher AKI incidence with platinum-based HIPEC than with mitomycin C–based treatment, with dialysis-requiring AKI occurring only in the cisplatin group [1]. These findings emphasize that renal risk should be considered before surgery, particularly in patients receiving cisplatin-based HIPEC.

AKI after CRS-HIPEC is best understood as the consequence of multiple interacting insults, including hemodynamic stress, systemic inflammation, endothelial and tubular injury, reduced physiological reserve, and cisplatin-related nephrotoxicity [6,10,14,15]. These mechanisms may be particularly relevant in patients with cancer, in whom immune dysfunction and nutritional impairment may coexist despite apparently preserved kidney function. This pathophysiological complexity provides a rationale for evaluating composite indices that capture inflammatory, immune, and nutritional status. Within this framework, patients who developed AKI had lower preoperative lymphocyte counts. Lymphopenia may reflect cancer-related immune dysfunction, systemic stress, reduced adaptive immune reserve, and the relative predominance of neutrophil-mediated inflammatory responses, all of which may be associated with greater susceptibility to perioperative organ injury [16,23]. Given the potential relevance of lymphocyte-related immune status, we evaluated several composite indices incorporating lymphocyte count together with inflammatory, hematologic, or nutritional parameters.

The most novel finding of our study was the association between a lower preoperative CALLY index and postoperative AKI after CRS-HIPEC. Its discriminatory performance, with an AUC of 0.708, was numerically higher than that of lymphocyte count alone and the other lymphocyte-based composite indices, suggesting that the additional contributions of CRP and albumin may provide information beyond the shared lymphocyte-related signal. Its potential advantage lies in integrating three complementary domains: systemic inflammation reflected by CRP, nutritional and hepatic synthetic status reflected by albumin, and adaptive immune status reflected by lymphocyte count [18,19]. A low CALLY index may therefore identify patients whose baseline kidney function appears preserved but whose overall capacity to tolerate perioperative stress is reduced. A CALLY cutoff of ≤1.640 yielded 79.2% sensitivity and 63.5% specificity for postoperative AKI. These findings indicate moderate discrimination and do not support the use of CALLY as a standalone preoperative marker. Rather, it may serve as an exploratory marker of systemic vulnerability associated with postoperative AKI and may support closer perioperative renal surveillance, careful hemodynamic management, avoidance of unnecessary nephrotoxic exposure, and early postoperative monitoring of kidney function. However, the data-derived threshold should be considered exploratory, as no internal or external validation was performed.

Baseline serum creatinine and eGFR have previously been associated with AKI after CRS-HIPEC [4,6,8]. In our cohort, however, neither measure differed between patients with and without postoperative AKI. This should not be interpreted as diminishing their value in renal risk assessment; rather, it may reflect the exclusion of patients with established CKD and the resulting narrow distribution of baseline kidney function. Most patients in our cohort were classified as ASA II or III, and nearly half had hypertension. In this setting, serum creatinine and eGFR remain essential measures of baseline kidney function but may not fully capture renal reserve or susceptibility to the combined hemodynamic, inflammatory, and nephrotoxic stresses of CRS-HIPEC. The associations observed with hypertension, lower hemoglobin levels, and AKI are consistent with previous studies by Hakeam et al. [5], Krause et al. [24], Angeles et al. [13], and a broader surgical report [25], and may reflect additional dimensions of vulnerability, whereas vasopressor use and postoperative mechanical ventilation may instead reflect perioperative instability and greater illness severity.

Consistent with previous studies [1,3,9,11,13], AKI was associated with substantially worse postoperative outcomes. Patients who developed AKI had higher in-hospital mortality, more postoperative complications, and longer ICU and hospital stays, suggesting that postoperative AKI was associated with clinically important adverse outcomes rather than representing only a biochemical increase in serum creatinine. Outcomes were particularly poor among patients with stage 3 AKI: six of eight died during hospitalization, all experienced at least one postoperative complication, and four required RRT. These stage-specific analyses were descriptive and underpowered to support reliable inference because of the small number of patients in each AKI stage. Although these stage-specific findings should be interpreted cautiously, they highlight the particularly unfavorable clinical course associated with severe AKI. Naffouje et al. similarly described AKI as a major complication of CRS-HIPEC closely associated with perioperative physiological stress and subsequent organ dysfunction [3]. Peak serum creatinine occurred at a median of postoperative day 3, consistent with the findings of Manna et al. [26], suggesting that renal function should be closely monitored during the first several postoperative days. Given that HIPEC-associated AKI may progress to acute kidney disease or CKD [6], several studies have reported the need for dialysis in the management of AKI [1,8,11,13]. These observations also support the importance of renal follow-up after discharge, particularly in patients with severe AKI.

This study has several limitations and strengths. Its retrospective, single-center design and modest sample size limit generalizability, while the inclusion of patients with different primary malignancies may have introduced heterogeneity in tumor biology, disease burden, previous treatment, and perioperative risk. The limited number of AKI events necessitated parsimonious regression models and restricted more comprehensive adjustment for confounders and stage-specific analyses. Accordingly, residual confounding related to hypertension, anemia, tumor burden, and other unmeasured or incompletely captured perioperative factors cannot be excluded. The data-derived threshold should not be used clinically without independent validation. AKI was defined using the serum creatinine–based KDIGO criteria because reliable urine output data were unavailable. In addition, potentially relevant intraoperative variables, including operative duration, estimated blood loss, fluid balance, and the use of goal-directed fluid therapy, were not systematically available for all patients; therefore, residual confounding related to hemodynamic and fluid-management factors cannot be excluded. Long-term renal outcomes were not assessed, precluding evaluation of progression from AKI to acute kidney disease or CKD. Detailed information on cumulative preoperative chemotherapy exposure, particularly prior platinum-based treatment, was also unavailable. Finally, because all patients received cisplatin plus mitomycin C during HIPEC, the findings cannot be extrapolated to other regimens or used to compare regimen-specific nephrotoxicity. Because 23% of patients had CRP values below 2.50 mg/L, the CRP floor was not an artefact of censoring. Nevertheless, the concentration of values near the lower reference range may have reduced the discriminatory contribution of the CRP component. The study also has several strengths. All procedures were performed by the same surgical team using a uniform dual-agent HIPEC regimen, reducing procedural and treatment-related heterogeneity. Patients with established CKD were excluded, serum creatinine was measured serially during the first postoperative week, and AKI was classified according to standardized KDIGO criteria. In addition, several inflammatory and immune-nutritional indices were evaluated within the same cohort, allowing direct comparison of their associations and discriminatory performance. To the best of our knowledge, this is the first study to evaluate the CALLY index in relation to AKI after CRS-HIPEC.

## 5. Conclusions

In this exploratory single-center cohort, a lower preoperative CALLY index (data-derived cutoff ≤ 1.640) was associated with postoperative AKI, yielding 79.2% sensitivity and 63.5% specificity. Among the evaluated inflammatory and immune-nutritional indices, the CALLY index had the numerically highest AUC, although its discriminative ability was moderate. As a simple measure derived from routinely available laboratory parameters, the CALLY index may serve as a candidate preoperative marker associated with postoperative AKI in patients undergoing CRS-HIPEC. These findings represent observational associations and should therefore be interpreted cautiously given the limited sample size, potential residual confounding, and lack of internal and external validation. Larger prospective multicenter studies with standardized perioperative data collection and long-term renal follow-up are needed to confirm these findings, validate the data-derived threshold in independent cohorts, and determine whether CALLY provides incremental information beyond established clinical and renal risk factors.

## Figures and Tables

**Table 1:** Preoperative and Intraoperative Characteristics of Patients According to Postoperative AKI Status.

Variable	Overall (n = 87)	Non-AKI (n = 63)	AKI (n = 24)	*p*-Value
Age (years), median [IQR]	60 [48–69]	61 [45–68]	59 [51–72]	0.294
Female sex, n (%)	65 (74.7)	47 (74.6)	18 (75.0)	1.000
BMI (kg/m^2^), mean ± SD	27.30 ± 4.83	27.56 ± 5.01	26.63 ± 4.35	0.398
BSA (m^2^), mean ± SD	1.79 ± 0.20	1.78 ± 0.19	1.80 ± 0.22	0.664
Primary tumor type				0.562
Colorectal	27 (31.0)	21 (33.3)	6 (25.0)	
Ovarian	25 (28.7)	19 (30.2)	6 (25.0)	
Gastric	13 (14.9)	9 (14.3)	4 (16.7)	
PMP	9 (10.3)	5 (7.9)	4 (16.7)	
Endometrial	6 (6.9)	4 (6.3)	2 (8.3)	
Mesothelioma	3 (3.4)	3 (4.8)	0 (0.0)	
Pancreatic	3 (3.4)	1 (1.6)	2 (8.3)	
Appendiceal	1 (1.1)	1 (1.6)	0 (0.0)	
ASA physical status				0.860
I	13 (14.9)	9 (14.3)	4 (16.7)	
II	44 (50.6)	33 (52.4)	11 (45.8)	
III	30 (34.5)	21 (33.3)	9 (37.5)	
ECOG performance status 2, n (%)	19 (21.8)	11 (17.5)	8 (33.3)	0.190
PCI, median [IQR]	10 [7–16]	10 [6–16]	11 [8–18]	0.617
Comorbidities				
Hypertension, n (%)	40 (46.0)	24 (38.1)	16 (66.7)	0.032
Diabetes mellitus, n (%)	19 (21.8)	14 (22.2)	5 (20.8)	1.000
Coronary artery disease, n (%)	9 (10.3)	5 (7.9)	4 (16.7)	0.253
Preoperative RAS blocker use, n (%)	20 (23.0)	12 (19.0)	8 (33.3)	0.275
Preoperative systemic chemotherapy, n (%)	40 (46.0)	27 (42.9)	13 (54.2)	0.481
Preoperative platinum exposure, n (%)	38 (43.7)	26 (41.3)	12 (50.0)	0.623
Intraoperative data				
Cisplatin dose (mg), median [IQR]	120 [100–128]	120 [100–125]	120 [100–130]	0.372
Cisplatin dose (mg/m^2^), median [IQR]	63.5 [58.8–69.9]	63.4 [58.7–68.3]	63.5 [59.5–71.1]	0.768
Perfusate volume (mL), median [IQR]	3500 [3000–4000]	3500 [3000–4000]	3500 [3000–3550]	0.591
Vasopressor use, n (%)	4 (4.6)	0 (0.0)	4 (16.7)	**0.005**

Abbreviations: AKI, acute kidney injury; ASA, American Society of Anesthesiologists; BMI, body mass index; BSA, body surface area; ECOG, Eastern Cooperative Oncology Group; IQR, interquartile range; PCI, peritoneal cancer index; PMP, pseudomyxoma peritonei; RAS, renin–angiotensin system; SD, standard deviation. Continuous variables are presented as mean ± SD or median [IQR], and categorical variables as n (%). Between-group comparisons were performed using Student’s *t*-test or the Mann–Whitney U test for continuous variables and the chi-square test or Fisher’s exact test for categorical variables. Statistically significant *p*-values are shown in bold.

**Table 2:** Preoperative Laboratory Parameters and Systemic Inflammatory and Immune-Nutritional Indices According to Postoperative AKI Status.

Variable	Overall Cohort (n = 87)	Non-AKI (n = 63)	AKI (n = 24)	*p*-Value
Baseline serum creatinine, mg/dL	0.69 [0.57–0.81]	0.68 [0.57–0.80]	0.75 [0.57–0.81]	0.601
eGFR, mL/min/1.73 m^2^	97.79 ± 19.42	98.73 ± 18.78	95.33 ± 21.23	0.496
Urea, mg/dL	27.5 [20.3–35.3]	26.2 [20.1–35.3]	30.3 [24.6–35.3]	0.161
Hemoglobin, g/dL	11.29 ± 1.94	11.61 ± 1.78	10.46 ± 2.13	**0.025**
White blood cell count, ×10^9^/L	6.79 ± 2.22	6.78 ± 2.14	6.81 ± 2.48	0.961
Neutrophil count, ×10^9^/L	4.23 [2.99–6.00]	4.15 [2.99–5.83]	4.93 [2.90–7.00]	0.518
Lymphocyte count, ×10^9^/L	1.46 [1.03–1.86]	1.66 [1.15–2.05]	1.16 [1.00–1.63]	**0.022**
Monocyte count, ×10^9^/L	0.56 [0.45–0.69]	0.55 [0.45–0.69]	0.59 [0.43–0.70]	0.704
Platelet count, ×10^9^/L	276 [205–391]	273 [223–375]	322 [189–446]	0.582
Albumin, g/dL	3.96 [3.53–4.21]	4.00 [3.63–4.20]	3.88 [2.83–4.21]	0.312
CRP, mg/L	2.50 [2.50–4.92]	2.50 [2.50–2.83]	2.50 [2.50–42.78]	**0.049**
Glucose, mg/dL	96 [86–114]	95 [87–116]	98 [84–109]	0.883
Sodium, mEq/L	139.06 ± 3.41	139.43 ± 2.88	138.08 ± 4.46	0.180
Potassium, mEq/L	4.25 [3.90–4.54]	4.25 [3.96–4.50]	4.25 [3.82–4.59]	0.711
Calcium, mg/dL	9.15 [8.73–9.38]	9.18 [8.79–9.41]	9.10 [8.42–9.31]	0.428
Systemic inflammatory and immune-nutritional indices				
NLR	2.93 [1.90–4.88]	2.62 [1.88–3.73]	4.88 [1.92–7.15]	**0.042**
PLR	180.7 [117.5–314.5]	179.6 [114.6–250.6]	300.2 [131.4–447.2]	0.054
SII	730.7 [425.3–1584.9]	722.3 [439.8–1224.5]	1501.9 [356.6–2989.8]	0.185
PIV	398.9 [190.0–939.3]	373.3 [201.2–668.8]	537.9 [142.7–2051.2]	0.303
SIRI	1.49 [0.86–2.92]	1.47 [0.86–2.22]	1.62 [1.11–6.01]	0.118
CALLY index	1.84 [0.98–2.76]	2.09 [1.28–2.94]	1.33 [0.06–1.61]	**0.003**

Abbreviations: AKI, acute kidney injury; CALLY, C-reactive protein–albumin–lymphocyte index; CRP, C-reactive protein; eGFR, estimated glomerular filtration rate; IQR, interquartile range; NLR, neutrophil-to-lymphocyte ratio; PIV, pan-immune-inflammation value; PLR, platelet-to-lymphocyte ratio; SD, standard deviation; SII, systemic immune-inflammation index; SIRI, systemic inflammation response index. Data are presented as mean ± SD or median [IQR]. Between-group comparisons were performed using Student’s *t*-test or the Mann–Whitney U test, as appropriate. Statistically significant *p*-values are shown in bold.

**Table 3:** Postoperative Characteristics and Outcomes According to Postoperative AKI Status.

Variable	Overall Cohort (n = 87)	Non-AKI (n = 63)	AKI (n = 24)	*p*-Value
Invasive mechanical ventilation, n (%)	9 (10.3)	2 (3.2)	7 (29.2)	**0.001**
Aminoglycoside exposure, n (%)	4 (4.6)	1 (1.6)	3 (12.5)	0.062
Intravenous contrast exposure, n (%)	14 (16.1)	9 (14.3)	5 (20.8)	0.677
Renal replacement therapy, n (%)	4 (4.6)	0 (0.0)	4 (16.7)	**0.005**
Any complication, n (%)	33 (37.9)	18 (28.6)	15 (62.5)	**0.008**
ICU stay (days), median [IQR]	2 [1–2]	1 [1–2]	2.5 [1.0–6.3]	**0.002**
Total hospital stay (days), median [IQR]	11 [8–17]	10 [7.5–14.5]	15.5 [10–31]	**0.016**
In-hospital mortality, n (%)	7 (8.0)	1 (1.6)	6 (25.0)	**0.002**

Abbreviations: AKI, acute kidney injury; ICU, intensive care unit; IQR, interquartile range; Renal replacement therapy included intermittent hemodialysis and/or continuous renal replacement therapy. Postoperative complications included bleeding, wound infection, anastomotic leakage, intra-abdominal abscess, evisceration, and bladder fistula. Continuous variables were presented as median [IQR], and categorical variables as n (%). Between-group comparisons were performed using Student’s *t*-test or the Mann–Whitney U test for continuous variables and the chi-square test or Fisher’s exact test for categorical variables. Statistically significant *p*-values are shown in bold.

**Table 4:** Clinical Characteristics and Outcomes According to Postoperative AKI Stage.

Variable	Non-AKI (n = 63)	AKI Stage 1 (n = 12)	AKI Stage 2 (n = 4)	AKI Stage 3 (n = 8)	*p*-Value
Peak serum creatinine, mg/dL	0.67 [0.56–0.82]	1.12 [0.81–1.38]	1.49 [1.37–1.63]	2.95 [1.49–4.62]	**<0.001 †**
ICU stay, days	1.0 [1.0–2.0]	1.5 [1.0–2.2]	2.0 [1.8–3.2]	7.5 [5.8–17.0]	**0.004 †**
Hospital stay, days	10.0 [7.5–14.5]	12.0 [7.0–14.8]	15.5 [12.5–19.2]	33.0 [28.0–42.5]	**0.017 †**
Outcomes, n (%) [95% CI]					
RRT	0 (0.0) [0.0–5.7]	0 (0.0) [0.0–26.5]	0 (0.0) [0.0–60.2]	4 (50.0) [15.7–84.3]	**<0.001 ‡**
In-hospital mortality	1 (1.6) [0.0–8.5]	0 (0.0) [0.0–26.5]	0 (0.0) [0.0–60.2]	6 (75.0) [34.9–96.8]	**<0.001 ‡**
Any complication	18 (28.6) [17.9–41.3]	4 (33.3) [9.9–65.1]	3 (75.0) [19.4–99.4]	8 (100.0) [63.1–100]	**<0.001 ‡**

Abbreviations: AKI, acute kidney injury; CI, confidence interval; ICU, intensive care unit; IQR, interquartile range; RRT, renal replacement therapy. Renal replacement therapy included intermittent hemodialysis and/or continuous renal replacement therapy. Postoperative complications included bleeding, wound infection, anastomotic leakage, intra-abdominal abscess, evisceration, and bladder fistula. Continuous variables presented as median [IQR]. Proportions were presented as n (%) with Clopper–Pearson exact 95% CIs. † Kruskal–Wallis test across all four groups. ‡ Fisher–Freeman–Halton exact test across all four groups. Statistically significant *p*-values are shown in bold.

**Table 5:** Age- and Sex-Adjusted, and Age-, Sex-, and Baseline eGFR-Adjusted Logistic Regression Analyses of Factors Associated with Postoperative AKI.

	Univariable								
				Model 1 Age- and Sex-Adjusted			Model 2 Age-, Sex-, and Baseline eGFR-Adjusted		
Variable	OR	95% CI	*p*-Value	Adj. OR	95% CI	*p*-Value	Adj. OR	95% CI	*p*-Value
Age (per year)	1.017	0.981–1.054	0.361						
Male sex	0.979	0.331–2.895	0.970						
BMI (per kg/m^2^)	0.960	0.869–1.061	0.421						
PCI (per unit)	1.026	0.961–1.094	0.447						
ECOG 2	2.364	0.811–6.886	0.115						
Hypertension	3.250	1.208–8.741	**0.020**	3.692	1.131–12.052	**0.030**	3.691	1.131–12.051	**0.031**
Diabetes mellitus	0.921	0.292–2.910	0.889						
Coronary artery disease	2.320	0.567–9.499	0.242						
Preoperative RAS blocker use	2.083	0.724–5.995	0.174						
Preoperative chemotherapy	1.576	0.612–4.056	0.346						
Preoperative platinum agent	1.423	0.553–3.659	0.464						
Cisplatin dose (per mg/m^2^)	1.007	0.961–1.056	0.768						
Perfusate volume (per mL)	1.000	1.000–1.001	0.458						
Baseline creatinine (per mg/dL)	1.299	0.093–18.065	0.845						
Baseline eGFR (per mL/min/1.73 m^2^)	0.991	0.967–1.016	0.465						
Hemoglobin (per g/dL)	0.734	0.569–0.946	**0.017**	0.736	0.569–0.953	**0.020**	0.717	0.546–0.941	**0.017**
Lymphocyte (per 0.1 × 10^9^/L)	0.901	0.814–0.997	**0.043**	0.903	0.815–1.001	0.053	0.901	0.811–1.000	**0.050**
Neutrophil (per ×10^9^/L)	1.046	0.894–1.225	0.572						
Monocyte (per ×10^9^/L)	2.277	0.315–16.446	0.415						
Platelet (per ×10^9^/L)	1.002	0.999–1.005	0.177						
Albumin (per g/dL)	0.450	0.218–0.929	**0.031**	0.426	0.201–0.904	**0.030**	0.433	0.198–0.947	**0.040**
CRP (per mg/L)	1.022	1.000–1.044	**0.047**	1.023	1.001–1.046	**0.044**	1.022	1.000–1.045	0.054
Glucose (per mg/dL)	1.003	0.992–1.015	0.591						
Urea (per mg/dL)	1.016	0.982–1.050	0.358						
Sodium (per mEq/L)	0.890	0.772–1.025	0.105						
Potassium (per mEq/L)	1.154	0.457–2.917	0.761						
Calcium (per mg/dL)	0.708	0.341–1.469	0.354						
NLR (per 1 unit)	1.183	0.987–1.417	0.069	1.205	1.001–1.450	**0.049**	1.199	0.996–1.443	0.055
PLR (per 10 units)	1.029	1.002–1.058	**0.037**	1.032	1.004–1.061	**0.027**	1.032	1.003–1.061	**0.028**
SII (per 100 units)	1.041	1.006–1.076	**0.020**	1.047	1.011–1.084	**0.010**	1.046	1.010–1.083	**0.011**
PIV (per 100 units)	1.032	0.999–1.066	0.056	1.037	1.003–1.073	**0.033**	1.036	1.002–1.072	**0.039**
SIRI (per1-unit increase)	1.132	0.959–1.337	0.143						
CALLY index (per 1-unit increase in log10 CALLY)	0.591	0.409–0.854	**0.005**	0.276	0.113–0.673	**0.005**	0.283	0.116–0.691	**0.006**

Abbreviations: AKI, acute kidney injury; BMI, body mass index; CALLY, C-reactive protein–albumin–lymphocyte index; CI, confidence interval; CRP, C-reactive protein; ECOG, Eastern Cooperative Oncology Group; eGFR, estimated glomerular filtration rate; NLR, neutrophil-to-lymphocyte ratio; OR, odds ratio; PCI, peritoneal cancer index; PIV, pan-immune-inflammation value; PLR, platelet-to-lymphocyte ratio; RAS, renin–angiotensin system; SII, systemic immune-inflammation index; SIRI, systemic inflammation response index. Model 1 was adjusted for age and sex. Model 2 was adjusted for age, sex, and baseline eGFR. Each variable of interest was evaluated in a separate adjusted logistic regression model; the candidate predictors were not entered together in a single model. Statistically significant *p*-values are shown in bold. The OR for intraoperative vasopressor use could not be estimated because of complete separation, as all patients who required vasopressor support developed AKI.

**Table 6:** ROC Analysis of Preoperative Inflammatory, Immune-Nutritional, and Renal Parameters for Postoperative AKI.

Variable	AUC	95% CI	Cutoff	Sensitivity	Specificity	Youden J
CALLY index	0.708	0.579–0.836	≤1.640	0.792	0.635	0.427
Lymphocyte count, ×10^3^/µL	0.660	0.536–0.784	≤1.630	0.792	0.508	0.300
PLR	0.640	0.494–0.785	≥269.200	0.542	0.810	0.351
NLR	0.618	0.472–0.764	≥4.960	0.500	0.857	0.357
SII	0.612	0.456–0.769	≥2318.800	0.500	0.889	0.389
SIRI	0.597	0.448–0.745	≥2.844	0.500	0.825	0.325
PIV	0.583	0.424–0.741	≥1828.150	0.458	0.889	0.347
Baseline serum albumin (g/dL)	0.615	0.470–0.760	≤3.020	0.375	0.921	0.296
Baseline eGFR (mL/min/1.73 m^2^)	0.541	0.393–0.689	≤85.000	0.417	0.762	0.179
Baseline serum creatinine (mg/dL)	0.494	0.348–0.640	≥0.700	0.583	0.556	0.139

Abbreviations: AKI, acute kidney injury; AUC, area under the curve; CALLY, C-reactive protein–albumin–lymphocyte index; CI, confidence interval; eGFR, estimated glomerular filtration rate; NLR, neutrophil-to-lymphocyte ratio; PIV, pan-immune-inflammation value; PLR, platelet-to-lymphocyte ratio; ROC, receiver operating characteristic; SII, systemic immune-inflammation index; SIRI, systemic inflammation response index. Ninety-five percent confidence intervals were calculated using the DeLong method. The thresholds were data-derived exploratory cutoffs determined by maximizing the Youden index (sensitivity + specificity − 1). Lower values indicated higher AKI risk for the CALLY index, lymphocyte count, and eGFR, whereas higher values indicated higher risk for the remaining markers. The data-derived CALLY cutoff of ≤1.640 was calculated using albumin in g/dL, lymphocyte count in ×10^3^/µL, and C-reactive protein in mg/L.

## Data Availability

The datasets used during the study have not been made publicly available due to patient privacy but are available from the corresponding author upon reasonable request.

## Supplementary Materials

The following supporting information can be downloaded at https://www.mdpi.com/article/10.3390/jcm15187308/s1: Table S1: Model Fit Statistics for Covariate-Adjusted Logistic Regression Models; Table S2: Pairwise DeLong Comparisons of the AUC of the CALLY Index with Other Preoperative Parameters for Postoperative AKI.

## Abbreviations

The following abbreviations are used in this manuscript:

Acute kidney injury	AKI
Akaike information criterion	AIC
American Society of Anesthesiologists	ASA
Area under the curve	AUC
Body mass index	BMI
Chronic kidney disease	CKD
C-reactive protein–albumin–lymphocyte	CALLY
Cytoreductive surgery with hyperthermic intraperitoneal chemotherapy	CRS-HIPEC
Chronic Kidney Disease Epidemiology Collaboration	CKD-EPI
Confidence interval	CI
C-reactive protein	CRP
Cytoreduction	CC
Eastern Cooperative Oncology Group	ECOG
Estimated glomerular filtration rate	eGFR
Hemodialysis	HD
Intensive care unit	ICU
Interquartile range	IQR
Kidney Disease: Improving Global Outcomes	KDIGO
Neutrophil-to-lymphocyte ratio	NLR
Odds ratio	OR
Pan-immune-inflammation value	PIV
Peritoneal cancer index	PCI
Platelet-to-lymphocyte ratio	PLR
Receiver operating characteristic	ROC
Renal replacement therapy	RRT
Renin–angiotensin system	RAS
Systemic immune-inflammation index	SII
Systemic inflammation response index	SIRI
Standard deviation	SD

## References

[1] Tan G.H.C., Shannon N.B., Chia C.S., Soo K.C., Teo M.C.C. (2018). Platinum agents and mitomycin C-specific complications in cytoreductive surgery (CRS) and hyperthermic intraperitoneal chemotherapy (HIPEC). Int. J. Hyperth..

[2] Solanki S.L., Salunke B., Gangakhedkar G., Ambulkar R., Kuberkar D.V., Bhatt A. (2024). Acute kidney injury after cytoreductive surgery and hyperthermic intraperitoneal chemotherapy—A systematic review. Eur. J. Surg. Oncol..

[3] Naffouje S.A., Tulla K.A., Chorley R., Armstrong N., Salti G.I. (2018). Acute kidney injury increases the rate of major morbidities in cytoreductive surgery and HIPEC. Ann. Med. Surg..

[4] Carias E., Ferreira H., Chuva T., Paiva A., Maximino J. (2022). Acute Kidney Injury After Cytoreductive Surgery and Hyperthermic Intraperitoneal Chemotherapy in a Portuguese Population. World J. Oncol..

[5] Hakeam H.A., Breakiet M., Azzam A., Nadeem A., Amin T. (2014). The incidence of cisplatin nephrotoxicity post hyperthermic intraperitoneal chemotherapy (HIPEC) and cytoreductive surgery. Ren. Fail..

[6] Lu Y., Xiao Z., Zhao X., Ye Y., Li S., Guo F., Xue H., Zhu F. (2024). Incidence, risk factors, and outcomes of the transition of HIPEC-induced acute kidney injury to acute kidney disease: A retrospective study. Ren. Fail..

[7] Cheng C.C., Yeh H.C., Su P.W., Ho C.L., Chang S.C. (2024). Risk factors of chronic kidney disease in cisplatin-based hyperthermia intraperitoneal chemotherapy. Int. J. Hyperth..

[8] Sin E.I., Chia C.S., Tan G.H.C., Soo K.C., Teo M.C. (2017). Acute kidney injury in ovarian cancer patients undergoing cytoreductive surgery and hyperthermic intra-peritoneal chemotherapy. Int. J. Hyperth..

[9] Arjona-Sánchez A., Cadenas-Febres A., Cabrera-Bermon J., Muñoz-Casares F.C., Casado-Adam A., Sánchez-Hidalgo J.M., López-Andreu M., Briceño-Delgado J., Rufián-Peña S. (2016). Assessment of RIFLE and AKIN criteria to define acute renal dysfunction for HIPEC procedures for ovarian and nonovarian peritoneal malignances. Eur. J. Surg. Oncol..

[10] Chen C.Y., Chang H.Y., Lu C.H., Chen M.C., Huang T.H., Lee L.W., Liao Y.S., Chen V.C., Huang W.S., Ou Y.C. (2020). Risk factors of acute renal impairment after cytoreductive surgery and hyperthermic intraperitoneal chemotherapy. Int. J. Hyperth..

[11] Ye J., Ren Y., Wei Z., Peng J., Chen C., Song W., Tan M., He Y., Yuan Y. (2018). Nephrotoxicity and long-term survival investigations for patients with peritoneal carcinomatosis using hyperthermic intraperitoneal chemotherapy with cisplatin: A retrospective cohort study. Surg. Oncol..

[12] Beltrán D., Pintado M.C., Oñoro A., Unzúe I.L., Sanz R.G., Alonso M.D., Ortega M.A., de Mon M.Á., Losada E.N., Calvo A.G. (2024). Side-Effects on the Renal function of Cytoreductive Surgery with Hyperthermic Intraperitoneal Chemotherapy. Int. J. Med. Sci..

[13] Angeles M.A., Quenet F., Vieille P., Gladieff L., Ruiz J., Picard M., Migliorelli F., Chaltiel L., Martínez-Gómez C., Martinez A. (2019). Predictive risk factors of acute kidney injury after cytoreductive surgery and cisplatin-based hyperthermic intra-peritoneal chemotherapy for ovarian peritoneal carcinomatosis. Int. J. Gynecol. Cancer.

[14] Miller R.P., Tadagavadi R.K., Ramesh G., Reeves W.B. (2010). Mechanisms of Cisplatin nephrotoxicity. Toxins.

[15] Coccolini F., Corbella D., Finazzi P., Brambillasca P., Benigni A., Prussiani V., Ceresoli M., Manfredi R., Poiasina E., Bertoli P. (2016). Time course of cytokines, hemodynamic and metabolic parameters during hyperthermic intraperitoneal chemotherapy. Minerva Anestesiol..

[16] Bi J.B., Zhang J., Ren Y.F., Du Z.Q., Wu Z., Lv Y., Wu R.Q. (2020). Neutrophil-to-lymphocyte ratio predicts acute kidney injury occurrence after gastrointestinal and hepatobiliary surgery. World J. Gastrointest. Surg..

[17] Zhang Y., Tang M., Gu Q.H., Zhou L.N., Chen M.B. (2025). The Prognostic Value of Combined Systemic Immune-Inflammatory Index (SII) and Prognostic Nutritional Index (PNI) in Solid Tumor. Cancer Manag. Res..

[18] Aoyama T., Hashimoto I., Maezawa Y., Hara K., Kazama K., Komori K., Numata M., Tamagawa A., Fukuda M., Cho H. (2024). CRP-albumin-lymphocyte (CALLY) Index Is an Independent Prognostic Factor for the Esophageal Cancer Patients Who Received Curative Treatment. Anticancer Res..

[19] Nakashima K., Haruki K., Kamada T., Takahashi J., Tsunematsu M., Ohdaira H., Furukawa K., Suzuki Y., Ikegami T. (2024). Usefulness of the C-Reactive Protein (CRP)-Albumin-Lymphocyte (CALLY) Index as a Prognostic Indicator for Patients with Gastric Cancer. Am. Surg..

[20] Yu R., Song H., Bi Y., Meng X. (2022). Predictive role of the neutrophil: Lymphocyte ratio in acute kidney injury associated with off-pump coronary artery bypass grafting. Front. Surg..

[21] Inker L.A., Eneanya N.D., Coresh J., Tighiouart H., Wang D., Sang Y., Crews D.C., Doria A., Estrella M.M., Froissart M. (2021). New Creatinine- and Cystatin C-Based Equations to Estimate GFR without Race. N. Engl. J. Med..

[22] KDIGO Group (2012). KDIGO Clinical Practice Guideline for Acute Kidney Injury. Kidney Int. Suppl..

[23] Rabb H., Griffin M.D., McKay D.B., Swaminathan S., Pickkers P., Rosner M.H., Kellum J.A., Ronco C. (2016). Acute Dialysis Quality Initiative Consensus XIII Work Group. Inflammation in AKI: Current Understanding, Key Questions, and Knowledge Gaps. J. Am. Soc. Nephrol..

[24] Krause M., Mehdipour S., Veerapong J., Baumgartner J.M., Lowy A.M., Gabriel R.A. (2024). Development of a predictive model for risk stratification of acute kidney injury in patients undergoing cytoreductive surgery with hyperthermic intraperitoneal chemotherapy. Sci. Rep..

[25] Musallam K.M., Tamim H.M., Richards T., Spahn D.R., Rosendaal F.R., Habbal A., Khreiss M., Dahdaleh F.S., Khavandi K., Sfeir P.M. (2011). Preoperative anaemia and postoperative outcomes in non-cardiac surgery: A retrospective cohort study. Lancet.

[26] La Manna G., Virzì S., Deraco M., Capelli I., Accorsi A., Dalmastri V., Comai G., Bonomi S., Grassi A., Selva S. (2006). Tubular dysfunction after peritonectomy and chemohyperthermic treatment with cisplatin. In Vivo.

