# Supplementary material for: Association of the Preoperative CALLY Index with Acute Kidney Injury After Cytoreductive Surgery and Hyperthermic Intraperitoneal Chemotherapy: A Comparison with Other Inflammatory and Immune-Nutritional Indices
Source: J Clin Med. 2026 Sep 20;15(18):7308. doi: 10.3390/jcm15187308 (PMC13607733; doi:10.3390/jcm15187308)
Supplement: Supplementary file 1 [file jcm-15-07308-s001.zip › jcm-4507079-supplementary.pdf]

Supplementary Table S1. Model Fit Statistics for Covariate-Adjusted Logistic Regression Models

Predictor	Model 1 AIC	Model 1 pseudo-(R^2)	Model 2 AIC	Model 2 pseudo-(R^2)
CALLY index	100.012	0.1022	101.752	0.1047
SII	101.742	0.0853	103.500	0.0877
PLR	103.778	0.0655	105.310	0.0700
PIV	103.763	0.0656	105.492	0.0682
NLR	104.943	0.0541	106.589	0.0575
SIRI	106.222	0.0416	107.887	0.0449

Abbreviations: AIC, Akaike Information Criterion; CALLY, C-reactive protein–albumin–lymphocyte index; eGFR, estimated glomerular filtration rate; NLR, neutrophil-to-lymphocyte ratio; PIV, pan-immune-inflammation value; PLR, platelet-to-lymphocyte ratio; SII, systemic immune-inflammation index; SIRI, systemic inflammation response index. Model 1 was adjusted for age and sex. Model 2 was adjusted for age, sex, and baseline eGFR. Each variable was evaluated in a separate logistic regression model. Lower AIC and higher McFadden pseudo-R^2 indicate better relative model fit within the compared models.

Supplementary Table S2. Pairwise DeLong Comparisons of the AUC of the CALLY Index with Other Preoperative Parameters for Postoperative AKI

Comparator	n	CALLY AUC	Comparator AUC	ΔAUC	95% CI for ΔAUC	z	p-value	Holm-adjusted p-value
NLR	87	0.708	0.618	0.090	−0.031 to 0.211	1.457	0.145	0.686
PLR	87	0.708	0.640	0.068	−0.048 to 0.184	1.150	0.250	0.686
SIRI	87	0.708	0.597	0.111	−0.015 to 0.237	1.733	0.083	0.581
SII	87	0.708	0.612	0.095	−0.026 to 0.217	1.534	0.125	0.686
PIV	87	0.708	0.583	0.125	0.001 to 0.249	1.975	**0.048**	0.434
Lymphocyte count	87	0.708	0.660	**0.048**	−0.103 to 0.199	0.622	0.534	0.686
Albumin	87	0.708	0.615	0.093	−0.031 to 0.216	1.468	0.142	0.686
Baseline eGFR	87	0.708	0.541	0.167	−0.040 to 0.374	1.579	0.114	0.686
Baseline serum creatinine	87	0.708	0.494	0.213	0.007 to 0.419	2.027	0.043	0.426

Abbreviations: AKI, acute kidney injury; AUC, area under the receiver operating characteristic curve; CALLY, C-reactive protein–albumin–lymphocyte index; CI, confidence interval; eGFR, estimated glomerular filtration rate; NLR, neutrophil-to-lymphocyte ratio; PIV, pan-immune-inflammation value; PLR, platelet-to-lymphocyte ratio; SII, systemic immune-inflammation index; SIRI, systemic inflammation response index. ΔAUC was calculated as AUC (CALLY) − AUC (comparator). Pairwise comparisons were performed using two-sided paired DeLong tests for correlated ROC curves derived from the same 87 patients (24 with AKI and 63 without AKI). P-values were adjusted for multiple comparisons using the Holm procedure. Lower values indicated greater AKI risk for CALLY, lymphocyte count, albumin, and eGFR, whereas higher values indicated greater risk for the remaining markers. Statistically significant Holm-adjusted p-values are shown in bold.
